# Supplementary figures and images for: Mycobacterium tuberculosis response to cholesterol is integrated with environmental pH and potassium levels via a lipid metabolism regulator
Source: PLoS Genet. 2024 Jan 24;20(1):e1011143. doi: 10.1371/journal.pgen.1011143 (PMC10843139; doi:10.1371/journal.pgen.1011143)

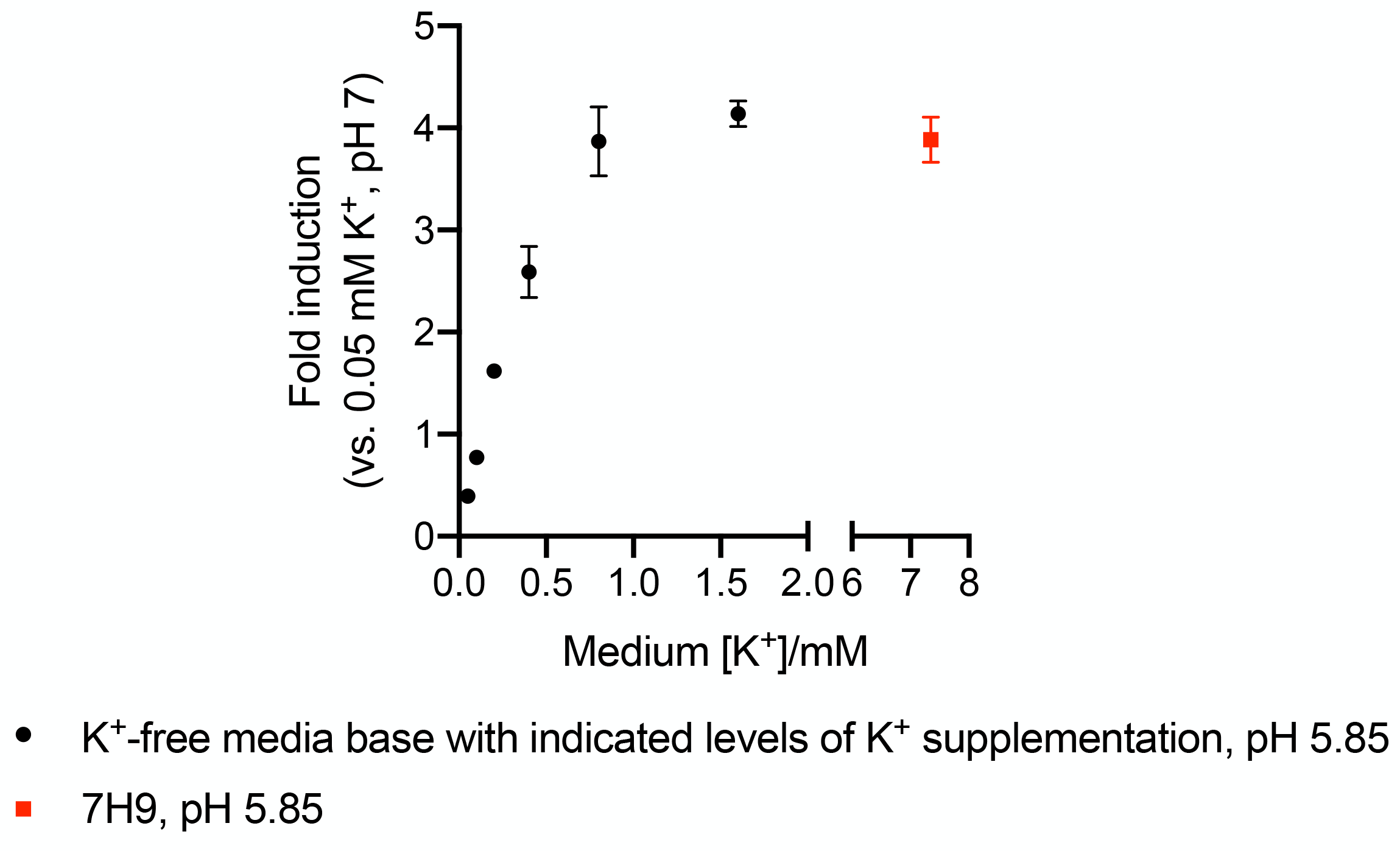

Supplement: S1 Fig — Mtb(rv2390c’::luciferase) was exposed to (i) K+-free 7H9, pH 7 medium supplemented with 0.05 mM K+, (ii) K+-free 7H9, pH 5.85 medium supplemented with indicated concentrations of K+, or (iii) 7H9, pH 5.85. 9 days post-exposure, light output (relative light units, RLU) and OD600 were measured. Fold induction compares RLU/OD600 in each condition to RLU/OD600 in the control 0.05 mM K+ 7H9, pH 7 condition. Data are shown as means ± SEM from four wells (two experiments). The numerical data underlying the graphs shown in this figure are provided in S1 Data. (TIF) [file pgen.1011143.s001.tif]

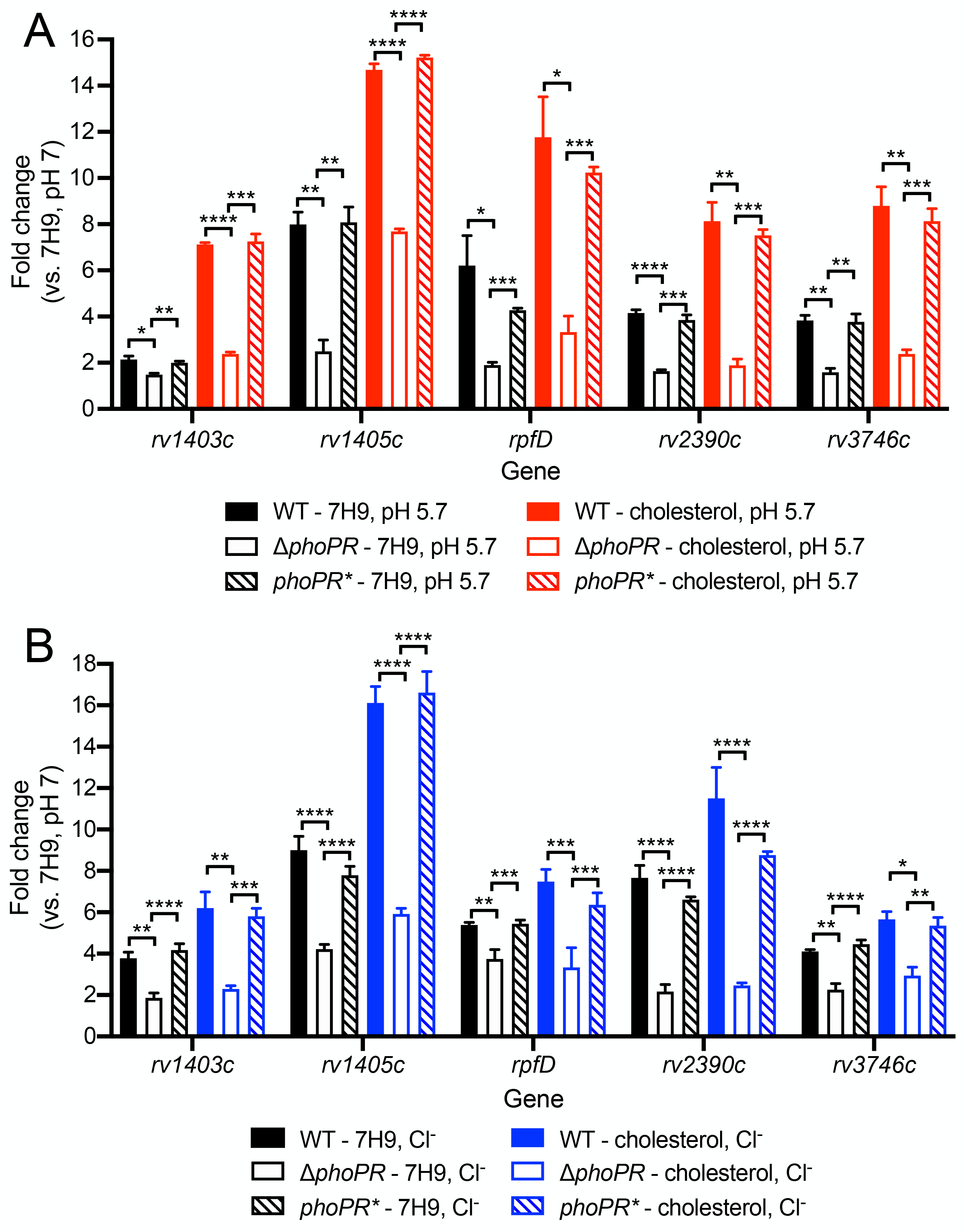

Supplement: S2 Fig — Log-phase WT, ΔphoPR, and phoPR* (complemented strain) Mtb were exposed for 4 hours to (A) 7H9 or cholesterol media at pH 5.7, or (B) 7H9 or cholesterol media at pH 7 + 250 mM NaCl, along with 7H9, pH 7 as the control condition. Fold change is as compared to the 7H9, pH 7 condition in all cases. sigA was used as the control gene, and data are shown as means ± SEM from 3 experiments. p-values were obtained with an unpaired t-test with Welch’s correction and Holm-Sidak multiple comparisons. N.S. not significant, * p<0.05, ** p<0.01, *** p<0.001, **** p<0.0001. The numerical data underlying the graphs shown in this figure are provided in S1 Data. (TIF) [file pgen.1011143.s002.tif]

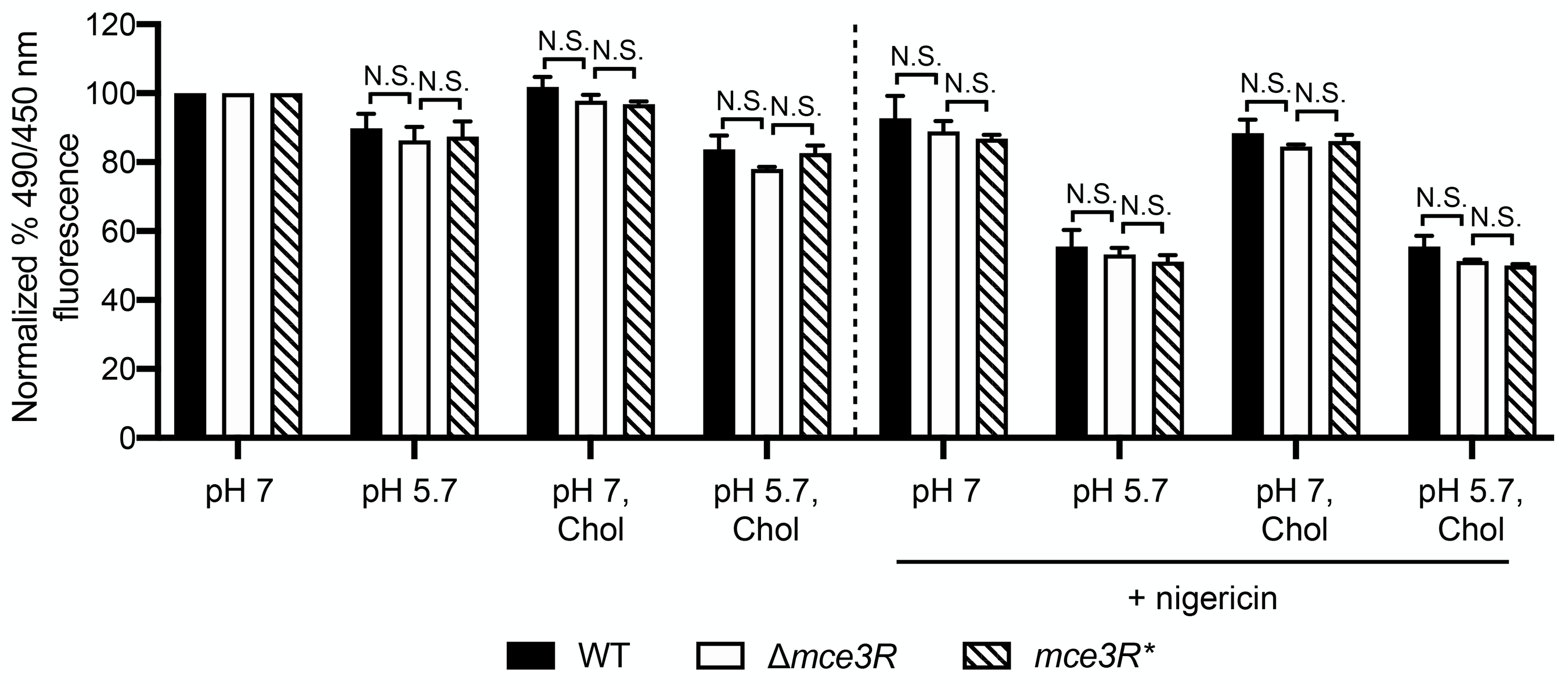

Supplement: S3 Fig — WT, Δmce3R, and mce3R* (complemented strain) Mtb were incubated for 1 hour with 5-chloromethylfluorescein diacetate, before washing and exposure to PBS at pH 7 or pH 5.7 containing 0.05% Tween 80, ± 0.2 mM cholesterol (“Chol”). For control samples, 10 μM nigericin was added to the respective buffers. Fluorescence at Ex. 450 nm and 490 nm, Em. 520 nm in both cases, was read on a microplate reader. Data were normalized as a percentage of the fluorescence ratios (Ex. 490 nm/Ex. 450 nm) observed in PBS, pH 7, for a given strain. Data are shown as means ± SEM from 3 experiments. p-values were obtained with a two-way ANOVA with Tukey’s multiple comparisons test. N.S. not significant. The numerical data underlying the graphs shown in this figure are provided in S1 Data. (TIF) [file pgen.1011143.s003.tif]

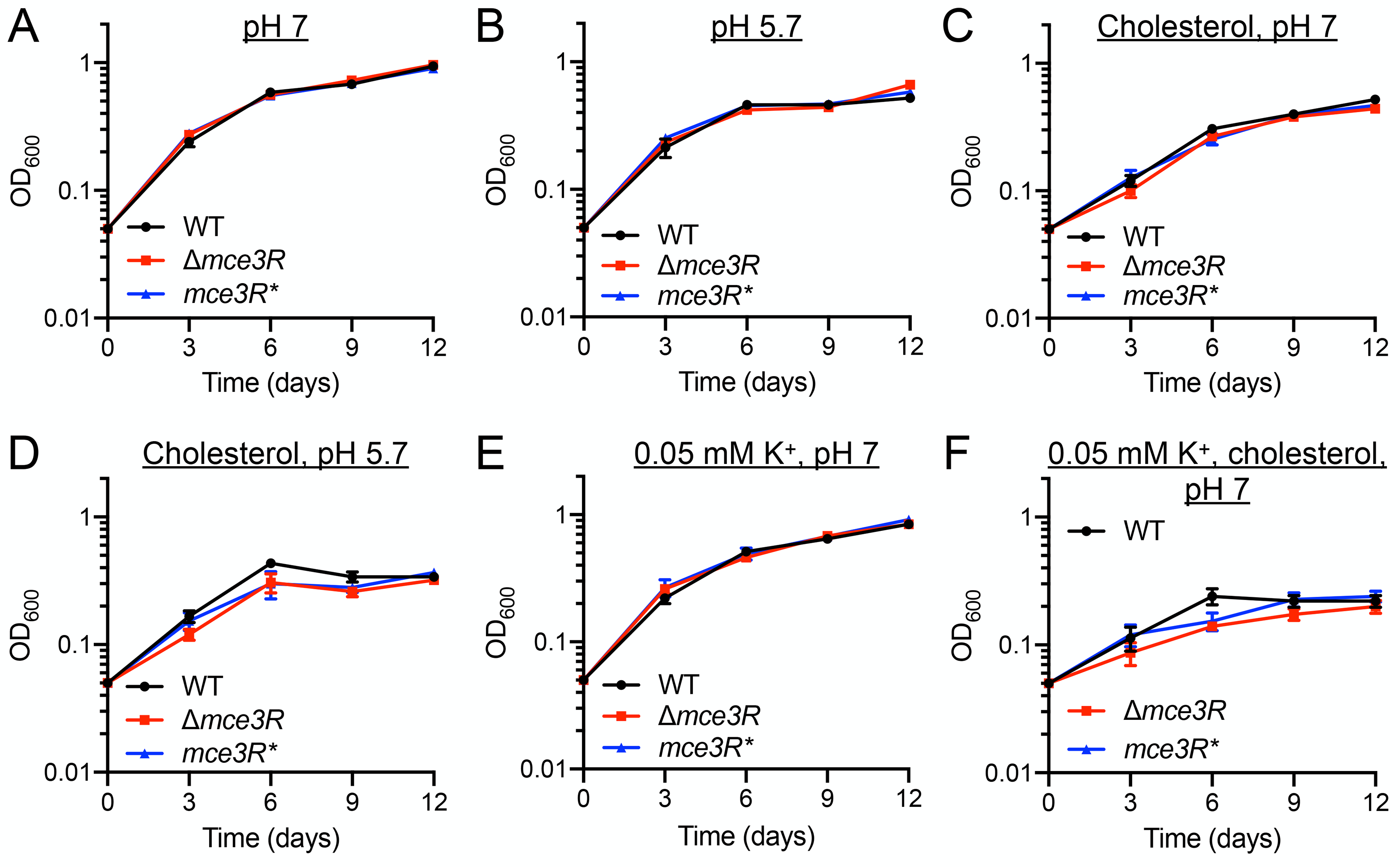

Supplement: S4 Fig — WT, Δmce3R and mce3R* (complemented strain) Mtb were grown in (A) 7H9, pH 7, (B) 7H9, pH 5.7, (C) cholesterol media, pH 7, (D) cholesterol media, pH 5.7, (E) K+-free 7H9, pH 7, supplemented with 0.05 mM K+, or (F) K+-free cholesterol media, pH 7, supplemented with 0.05 mM K+, and OD600 of the cultures tracked over time. Data are shown as means ± SEM from 3 experiments. The numerical data underlying the graphs shown in this figure are provided in S1 Data. (TIF) [file pgen.1011143.s004.tif]
